# Performance Investigation of High Temperature Application of Molten Solar Salt Nanofluid in a Direct Absorption Solar Collector

**DOI:** 10.3390/molecules24020285

**Published:** 2019-01-14

**Authors:** M. A. Karim, Owen Arthur, Prasad KDV Yarlagadda, Majedul Islam, Md Mahiuddin

**Affiliations:** 1 Science and Engineering Faculty, Queensland University of Technology, Brisbane QLD 4001, Australia; o.arthur@LIVE.com (O.A.); y.prasad@qut.edu.au (P.K.Y.); i.majedul@qut.edu.au (M.I.); md.mahiuddin@hdr.qut.edu.au (M.M.); 2 Department of Mechanical Engineering, Chittagong University of Engineering and Technology, Chittagong 4349, Bangladesh; 3 Department of Mechanical Engineering, Dhaka University of Engineering and Technology, Gazipur 1700, Bangladesh

**Keywords:** nanofluids, direct absorption solar collector, heat and mass transfer, computational fluid dynamics, molten salts

## Abstract

Nanofluids have great potential in a wide range of fields including solar thermal applications, where molten salt nanofluids have shown great potential as a heat transfer fluid (HTF) for use in high temperature solar applications. However, no study has investigated the use of molten salt nanofluids as the HTF in direct absorption solar collector systems (DAC). In this study, a two dimensional CFD model of a direct absorption high temperature molten salt nanofluid concentrating solar receiver has been developed to investigate the effects design and operating variables on receiver performance. It has been found that the Carnot efficiency increases with increasing receiver length, solar concentration, increasing height and decreasing inlet velocity. When coupled to a power generation cycle, it is predicted that total system efficiency can exceed 40% when solar concentrations are greater than 100×. To impart more emphasis on the temperature rise of the receiver, an adjusted Carnot efficiency has been used in conjunction with the upper temperature limit of the nanofluid. The adjusted total efficiency also resulted in a peak efficiency for solar concentration, which decreased with decreasing volume fraction, implying that each receiver configuration has an optimal solar concentration.

## 1. Introduction

The overall efficiency of a concentrating solar power system depends on three main factors, the efficiency of the receiver, field efficiency (effective capacity to theoretical capacity) and the Carnot efficiency. With the use of nanofluids both receiver and Carnot efficiencies can be improved [1,2]. Nanofluid heat transfer fluid will also have significant impact of thermal storage performance [3,4]. 

In a conventional solar thermal receiver the solar radiation is directed onto a high-absorptive surface where it is converted to thermal energy [5,6,7,8,9]. Spectrally selective surfaces are used to achieve both high absorptivity in the solar spectrum and low emissivity in the infrared [10,11,12,13,14,15]. The collected thermal energy is then transferred to a heat transfer fluid (HTF) to be used in a thermodynamic cycle. These surface based receivers, while being efficient at converting solar to thermal energy, suffer from two major drawbacks at high temperatures. First, the receiver surface being directly in contact with the environment has significant convective and radiative losses which increase with the temperature, also leading to a temperature difference between the surface and the fluid lowering the overall conversion efficiency. Second, the high temperatures cause significant thermal stress on the material causing it to degrade [16]. An alternative concept to avoid these drawbacks is to use a direct absorption collector (DAC) in place of the surface collector [17]. A DAC works by absorbing the solar radiation directly in the HTF, resulting in a more uniform distribution and a decrease in the temperature difference between the absorber and fluid. There exist numerous DAC designs, including the solar pond, trickle collectors, small particle collectors, volume trap collectors and black liquid collectors [17,18,19,20]. With the recent advancements in nanotechnology, small particle collectors have gained significant interest, with the small particles being of the nanometre scale the absorption of solar radiation can be significantly improved by low particle volume concentrations. Also due to their small size the particles are essentially fluidized, meaning they can pass through pumps, micro-channels and piping without any adverse effects [21]. Nanofluids provide a number of advantages in DAC including:

- The performance of the receiver can be tuned to suit conditions by altering the size, shape, solar concentration and material type of the nanoparticles, as the optical properties of the nanofluid are dominated by the nanoparticles [21,22].
- DACs do not require a surface absorption plate, which results in a significantly simpler receiver design and reduced cost and labour, as surface-absorbing plates require complex manufacturing processes [23]
- Nanofluids also possess superior thermophysical properties, such as enhanced thermal conductivity, heat transfer coefficient and in some cases enhanced specific heat capacity [23,24,25].

Tyagi et al. [26] developed a two dimensional heat transfer analysis of direct sunlight incident on a thin flowing film of water/aluminium nanofluid. Using a finite difference method they found an increase in efficiency of approximately 10% of the volumetric receiver compared to a conventional flat-plate solar collector. Without performing any experimental work, this theoretical study only considered direct sun and neglected in-scattering effect [26]. Otanicar et al. [27] extended on Tyagi et al. [26] work by investigating the properties of the base fluids, included size-dependent effects on the nanoparticle optical properties and verified the model experimentally. They found an increase in receiver efficiency of up to 5% by using nanofluids and that after a steep initial increase of the efficiency levels off as the volume fraction continues to increase [27]. Taylor et al. [28] conducted a conservative, simplified analysis of how a medium temperature (100–400 °C) nanofluid Concentrating Solar Power (CSP) system would perform compared to a conventional one. It was found that an efficiency improvement in the order of 5–10% was possible when using a nanofluid receiver. For a 100 MW nanofluid thermal plant, such an improvement in efficiency can equate to an addition of $3.5 million to the yearly revenue [21]. Experimental work was also conducted to validate the model; however, the results did not match well with the theoretical model. The discrepancies were attributed to the instability of the nanofluid (agglomeration and sedimentation) and the fact that more concentrated light will be absorbed in a thin upper layer of the nanofluid, which would be easily transferred back out of the receiver. Veeraragavan et al. [29] developed a non-dimensional analytical model to account for the effect of heat loss, particle loading, solar concentration and channel height on receiver efficiency with a Therminol VP-1 graphite nanofluid. The total system efficiency was determined by combining the receiver efficiency with the Carnot efficiency to determine an optimum value of 35% at a dimensionless receiver length of 0.86 [24]. Lenert and Wang [30] presented a combined modelling and experimental study to optimize the efficiency of liquid based solar receivers using carbon-coated absorbing nanoparticles. A transient one-dimensional heat transfer model and a cylindrical nanofluid volume receiver were used as the model and experiment respectively, and showed good agreement in results for varying optical thicknesses of the nanofluid. It was predicted that receiver-side efficiencies could exceed 35% when the receiver is optimized with respect to the optical thickness and solar exposure time [30]. Luo et al. [31] presented a simulation model and validated it with an experimental setup where the results were in accordance with each other. It was found that nanofluids could increase the collector efficiency by 2–25% compared to the base fluid [31]. Parvin, Nasrin and Alim [32] investigated the heat transfer performance and entropy generation of forced convection through a direct absorption collector. The heat transfer performance was enhanced by up to 31% and the collector efficiency was enhanced by more than two times [32]. Kaluri et al. [16] recently presented a three dimensional CFD model of a direct absorbing collector that took into account the effects of optical concentration, optical density of fluid, mass flowrate and thermal insulation on the receiver efficiency. An increase of up to 28% in receiver efficiency was observed.

There is a large amount of published literature on the thermophysical and rheological properties of low temperature nanofluids. These works mainly include aqueous and glycol based nanofluids with the temperatures generally less than 100 °C. There is also comprehensive literature on the possible mechanisms behind the changes in thermophysical and rheological properties, which have shown good agreement with the experimental works in some instances. Numerous works have also presented on empirical models with good results. Molten salt nanofluids have received very little attention in comparison. These have been shown to act differently to other nanofluids, as with the addition of nanoparticles in molten salt nanofluids show an increase in specific heat capacity while other nanofluids, including those that are aqueous and glycol based, show a decrease. Similarly, some research studies have been conducted on modelling CSP nanofluid receivers. However, these works have also been limited to low to medium temperature nanofluids, the majority of which are aqueous, and glycol based [33]. There is yet to be any study, which focuses specifically on the use of molten salt nanofluids as the HTF in DAC systems. This study aims to address this issue by developing a computational model of a receiver in order to determine an optimal receiver design for DAC systems using molten solar salt (NaNO_3_-KNO_3_) nanofluids as the HTF. The model has been validated by comparing to the results found in the literature. Factors of the receiver to be taken into account are the thermal re-radiation of the HTF to the environment, convective and conductive heat transfer with the environment, forced convection due to wind, volume fraction of nanoparticles and height of receiver. This research is therefore significant because molten salt nanofluids have been shown as an HTF for high temperature applications. 

The paper is structured as follows: a comprehensive literature is reviewed and the research gap is identified in the introduction above, details of the computational model, including governing equations, solution method, initial and boundary conditions, model validation, and results and discussion is presented next. Finally, conclusion of this new study has been presented at the end.

## 2. Model Development

### 2.1. Model Setup

**Figure 1:**
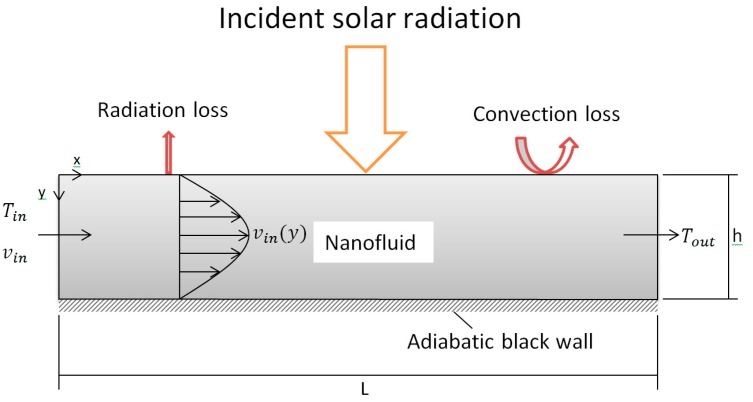
Model setup.

This study investigates a flowing solar receiver in which the concentrated solar radiation is assumed to be incident normally and directly absorbed within the channel due to suspended nanoparticles, as illustrated in Figure 1. The nanofluid is modelled using CFD module of COMSOL Multiphysics^®^ engineering software (4.3, COMSOL Inc., Burlington, MA, USA), which is assumed to be volumetrically heated as it flows between two parallel plates of height h. The model is 3D to account for a volumetric heat source but is 2D in nature as the width of the receiver does not affect the outcome of the model in any way and the sidewalls are modelled as planes of symmetry. The flow is modelled as fully developed with the two plates as no-slip walls and three heat losses are included, namely convection to the ambient, surface to ambient radiation and thermal re-emission from within the fluid, the first two of which act on the top surface. As shown in Equation (1), the thermophysical properties of the nanofluid are taken as those of the base fluid due to low concentrations of particles considered and are modelled as temperature dependent; the rheological properties are estimated by using Krieger type viscosity model. The bottom of the receiver is modelled as an adiabatic black wall and reflective losses are not considered.

Krieger-Dougherty model:

(1) $$\mu_{nf}=\mu_{bf}{\left(1-\frac{\phi_{a}}{\phi_{m}}\right)}^{1\left[\eta \right]\phi_{m}}$$

### 2.2. Modelling Optical Properties of the Nanofluid

The optical properties of nanofluids are dependent on a number of factors, namely, nanoparticle size, material type, shape and volume fraction. Different types of material are quantified by the complex refractive index, which is a combination of the refractive and absorptive indexes [34]:

(2) $$s_{np}=n_{np}+ia_{np}$$

The effects of particle shape are complicated and difficult to quantify. Currently there is no reliable theory that can be used to describe the impact of varying shape of nanoparticles [35]. Reliable theories exist for spherical particles and as such, assumptions will be made that all nanoparticles are spherical in shape. The effect of particle size is expressed through a size parameter, α, which is defined as follows:

(3) $$\alpha =\frac{\pi \;D}{\lambda }$$

To account for the absorption and scattering of spheres, the Mie theory is used [36,37]. While Mie theory provides a first-order description of optical effects in non-spherical particles and correctly describes many small-particle effects that are not intuitively obvious; the math itself is quite cumbersome. Therefore for simplicity, if the diameter of a particle is small when compared to the wavelength of light in a medium then Rayleigh type scattering can be assumed. Due to the incredibly small size ratio, many of the higher order components in Mie scattering theory can be ignored [36,37]:

(4) |m|α<<1

The extinction, scattering and radar backscattering efficiencies for Rayleigh scattering are given in Equations (5)–(7), respectively [36]:

(5) Qext=4αIm{m2−1m2+2[1+α215(m2−1m2+2)m4+27m2+382m2+3]}+83α4Re{(m2−1m2+2)2}

(6) $$Q_{scat}=\frac{8}{3}\alpha^{4}{\left|\frac{m^{2}-1}{m^{2}+2}\right|}^{2}$$

(7) $$Q_{b}=4\alpha^{4}{\left|\frac{m^{2}-1}{m^{2}+2}\right|}^{2}$$

If |m|α<<1, the coefficient of $\frac{m^{2}-1}{m^{2}+2}$ in the absorption efficiency equation is approximately unity [36], therefore the absorption efficiency reduces to Equation (8):

(8) Qabs=4αIm{m2−1m2+2}[1−4α33Im{m2−1m2+2}2]

(9) $$Q_{ext}=Q_{abs}+Q_{scat}$$

Furthermore, if 4α33Im{m2−1m2+2}<<1, which will occur for sufficiently small α, the absorption efficiency is reduced again to Equation (10) [36]:

(10) Qabs=4αIm{m2−1m2+2}

Additionally the extinction efficiency can be reduced to just the absorbing as it can be seen in the above equations when comparing *Q_abs_* and *Q_scat_* that *α*^4^ << *α*. This is due to nanoparticles being significantly smaller than the wavelengths, meaning that *α* < 1 resulting in *α*^4^ << *α*; therefore scattering effects can be ignored [37]. This simplification is only valid for sufficiently uniform small particles, as can be seen in the relationship shown in Equation (11) for the fraction of incident light that is scattered [28,36]. In addition, it should be noted that for volume fractions less than 0.6% dependent scattering effects can be ignored [38]:

(11) $$\frac{I_{s}}{I_{0}}\approx \frac{\pi^{4}ND^{6}}{8\lambda^{4}r^{2}}{\left|\frac{m^{2}-1}{m^{2}+2}\right|}^{2}\left(1+\cos^{2}\vartheta \right)$$

The extinction coefficient of the nanoparticles can then be represented as follows:

(12) $$\kappa_{np}=\frac{3}{2}\frac{\phi \left(Q_{abs}+Q_{scat}\right)}{D}$$

Under the assumption that no aggregation occurs the extinction coefficient becomes:

(13) $$\kappa_{np}=\frac{3}{2}\frac{\phi Q_{abs}}{D}$$

These equations assume that the base fluid is completely transparent, while this is desirable, in reality it is not the case. To account for this, the total extinction coefficient is proposed as the addition of the particles and the base fluid’s extinction coefficient [28]:

(14) $$\kappa =\kappa_{np}+\kappa_{bf}$$

(15) $$\kappa_{bf}=\frac{4\pi \alpha_{bf}}{\lambda }$$

As the incident radiation in question is concentrated solar radiation, it can be approximated using Planck’s black body distribution:

(16) $$I_{0}\left(\lambda \right)=S_{att}c\Omega_{s}\frac{2h_{p}c^{2}}{\lambda^{5}}\frac{1}{e^{\frac{hc}{\lambda k_{B}T_{sun}}}-1}$$

In the spectrum of the solar radiation outside of Earth’s atmosphere, 96.3% of the total irradiance is in the range of 200–2500 nm, and this range will be considered in this study.

### 2.3. Governing Equations 

Two physics modules are incorporated to take into account both the heat transfer and the fluid dynamics of the receiver. The heat transfer uses the following governing equation:

(17) $$\rho C_{p}u\cdot \nabla T=\nabla \cdot \left(k\nabla T\right)+Q$$

The fully developed velocity profile is determined taking into account particles being suspended in the fluid. The governing equations for the mixture model are listed below:

(18) $$\rho \left(u\cdot \nabla \right)u=\nabla \cdot \left[-pl+\mu \left(\nabla u+{\left(\nabla u\right)}^{T}\right)\right]-\nabla \cdot \left[\rho c_{d}\left(1-c_{d}\right)u_{slip}u_{slip}\right]+\rho_{c}\left(\nabla \cdot u\right)$$

(19) $$\nabla \cdot N_{\phi_{d}}=-\frac{m_{dc}}{\rho_{d}}$$

(20) $$N_{\phi_{d}}=\phi_{d}u_{d}$$

(21) $$u_{d}=u+\left(1-c_{d}\right)u_{slip}$$

(22) $$\rho =\phi_{c}\rho_{c}+\phi_{d}\rho_{d}$$

(23) $$c_{d}=\frac{\phi_{d}\rho_{d}}{\rho }$$

(24) $$3\frac{C_{d}\rho_{c}}{4d_{d}}\left|u_{slip}\right|u_{slip}=\frac{-\left(\rho -\rho_{d}\right)}{\rho }\nabla p$$

(25) Rep=ddρc|uslip|μ

(26) Cd=24Rep

In a number of previous studies plug flow was used in place of fully developed flow [20,28,39], under the assumption of a creeping flow, where the Reynolds number is mostly less than 1, or an inviscid flow. This assumption, however, does not hold for volumetric receivers, where the Reynolds number is generally between 10 and 1000 [28,39]. As such fully developed flow has been used in this study to overcome this limitation. To model the slip velocity between the nanoparticles and the base fluid the Hadamard-Rybcynski model is used.

To simplify the model, it is assumed that there is no temperature difference between the particles and the base fluid because the high surface to volume ratio of the nanoparticles leads to instantaneous heat transfer between the two mediums [40].

The absorbed thermal radiation is modelled as a volumetric heat release assuming that the change in normally incident spectral flux due to attenuation by the nanofluid is dissipated as a local heat release. The heat release function is determined from the radiative transfer equation [37]:

(27) $$\cos\theta \frac{dI_{\lambda }}{dy}=-\left(\kappa_{\lambda }+\sigma_{\lambda }\right)I_{\lambda }+\kappa_{\lambda }n_{\lambda }^{2}I_{b\lambda }\left(T\right)+\frac{\sigma_{\lambda }}{2}\int_{0}^{\pi }I_{\lambda }\left(y,\theta^{\prime }\right)p_{\lambda }\left(\theta^{\prime },\theta \right)\sin\theta^{\prime }d\theta^{\prime }$$

Given that the solar radiation is normally incident, scattering is negligible and the radiative transfer equation is in its quasi-steady form of the heat release function is defined as Equation (28) [37]. The quasi-steady assumption is valid, as the model will only be considered under steady state conditions:

(28) $$\frac{dI_{\lambda }}{dy}=\kappa_{\lambda }\left(I_{b\lambda }\left(T\right)-I_{\lambda }\right)$$

where, *y* is the distance from the top of the receiver. Integrating both sides yields:

(29) $$I_{\lambda }\left(y\right)=I_{0,\lambda }e^{-\kappa_{\lambda }y}+I_{b\lambda }\left(T\right)\left(1-e^{-\kappa_{\lambda }y}\right)$$

where, $I_{0,\lambda }$ is the concentrated normally incident solar radiation quantified using Planck’s black body distribution previously defined in this study. Differentiating with respect to y and integrated over the solar wavelength range the volumetric heat release is given as:

(30) $$Q\left(y\right)=-\int_{\lambda_{\min}}^{\lambda_{\max}}\kappa_{\lambda }\left(I_{b\lambda }\left(T\right)-I_{0,\lambda }\right)\;e^{-\kappa_{\lambda }y}dy$$

The top surface of the receiver consists of two types of heat losses, convective loss and radiative loss. For the heat losses to be combined, the radiative loss needs to be approximated as a linear heat loss coefficient, so that the two heat loss coefficients can be summed together. The radiative linear heat loss coefficient is a good approximation for low temperatures but loses the accuracy for high temperatures (T > 750 K) [20]. As this study is investigating high temperature situations, the heat losses will need to be considered separately. The radiative heat loss to the ambient is calculated using Stefan-Boltzmann’s law:

(31) $$q=\varepsilon \sigma \left(T_{amb}^{4}-T^{4}\right)$$

The convective heat loss is quantified using an overall heat transfer coefficient that takes into account convection between the nanofluid and the top surface as well as conduction through the receiver top plate:

(32) $$\frac{1}{h_{total}}=\frac{1}{h_{nf}}+\frac{t}{k_{tp}}$$

Fused quartz was chosen as the material for the top surface as it has a relatively low thermal conductivity of 1.3 (W/mK) and stability at high temperatures with an annealing point of 1140 °C. Fused quartz also possesses an extremely low coefficient of thermal expansion, reducing the effects of thermal shock and most importantly, it is virtually transparent in the solar range with an absorptive index ranging from 1.72 × 10^−6^–1.354 × 10^−5^ [39]. An arbitrary thickness of 10 mm was chosen for the top plate to achieve good insulation. The heat transfer coefficient of the nanofluid was determined using the average Nusselt number:

(33) $$h=\frac{Nuk}{L}$$

The average Nusselt number is given as follows:

(34) Nu=0.664Re12Pr13

The bottom surface of the receiver is considered to be an adiabatic black wall to the incident radiation, so any radiation that reaches the surface is completely absorbed and converted to heat. The absorbed heat is modelled as a heat flux by substituting the receiver height for y in Equation (28) and integrating over the solar range:

(35) $$P_{bottom,surface}=\int_{\lambda_{\min}}^{\lambda_{\max}}\left(I_{0,\lambda }e^{-\kappa_{\lambda }y_{rec}}+I_{b\lambda }\left(T\right)\left(1-e^{-\kappa_{\lambda }y_{rec}}\right)\right)d\lambda$$

Finally, the receiver efficiency is defined as the ratio of usable thermal energy to the incident solar energy:

(36) $$\eta_{rec}=\frac{\dot{m}C_{p}\left(T_{out}-T_{in}\right)}{I_{0}A_{r}}$$

Converting the receiver efficiency equation to 2D gives the following equation:

(37) $$\eta_{rec}=\frac{\rho_{bf}v_{in}y_{rec}w_{rec}C_{p,bf}\left(T_{out}-T_{in}\right)}{P_{y,0}l_{rec}w_{rec}}$$

### 2.4. Solution Procedure

**Figure 2:**
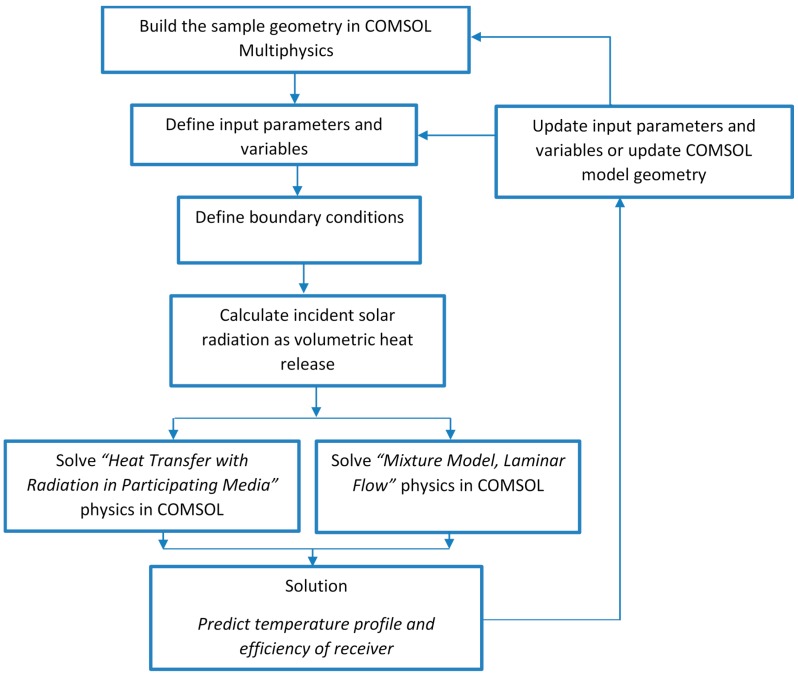
COMSOL Multiphysics modelling strategy.

The solution method of the governing equations using COMSOL Multiphysics modelling package is shown in Figure 2.

#### 2.4.1. Model Parameters and Variables

The values of the parameters presented in Table 1 were chosen to reflect those chosen by Veeraragavan et al. [29] for their model as the current model was also validated comparing with that study. Variables considered in the simulation are: the wavelength dependent refractive and absorptive indices of the nanoparticle, thermal re-radiation of the HTF to the environment, convective and conductive heat transfer with the environment, volume fraction of nanoparticles, size of nanoparticles, length of the receiver and height of the receiver as shown in Table 2.

#### 2.4.2. Initial and Boundary Conditions

The thermal insulation or adiabatic surface, symmetry and outflow boundary conditions were simulated as:

(38) −n⋅(−k∇T)=0

Opaque Surface:

(39) $$-n\cdot \left(-k\nabla T\right)=q_{r,net}$$

(40) $$I_{i,bnd}=\varepsilon_{W}{/}_{b}\left(T\right)+\frac{1-\varepsilon_{W}}{\pi }q_{r,net},n\cdot S_{i}<0$$

(41) $$q_{r,out}=\sum_{n\cdot S_{j}>0}\omega_{j}{/}_{j}n\cdot S_{j}$$

The temperature boundary condition was used to simulate the inlet temperature of the receiver:

(42) $$T=T_{0}$$

Heat Flux:

(43) $$-n\cdot \left(-k\nabla T\right)=h\cdot \left(T_{ext}-T\right)$$

#### 2.4.3. Grid Generation

**Figure 3:**
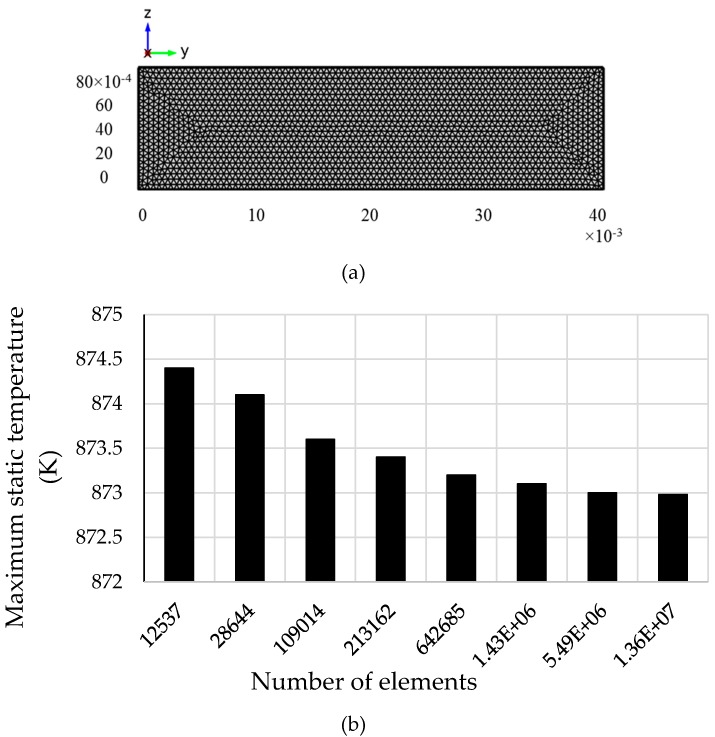
(**a**) Generated mesh for the Therminol model and (**b**) stability of the maximum static temperature of the receiver for different grid systems.

The grid system used in this study is presented in Figure 3a, which was then used in COMSOL simulations. A grid sensitivity test was first performed to determine the optimum grid system for the current study as presented in Figure 3b. Maximum static temperature (K) was recorded from the model for eight different grid systems as shown in the figure. From the figure, it is clear that grid system higher than 54,928,84 elements is quite sufficient to produce reasonably stable result for the current model. Hence, the results produced from this simulation study were totally grid independent.

#### 2.4.4. Thermo-Physical Properties of Nanofluid

This study considers a combination of sodium nitrate and potassium nitrate salts (NaNO_3_-KNO_3_) at a mole fraction of 60:40 respectively and is also commonly referred to as solar salt. This molten salt was chosen to be doped with graphite nanoparticles due to graphite’s high absorptivity in the solar range and its common occurrence in literature as a dopant. Moreover, molten salt nanofluid is very well suited to high temperature direct absorption solar collector systems. Very low volume concentrations of nanoparticles are considered (*f*_v_ < 0.006) in this study and therefore it is assumed that the particles will have negligible effects on the thermophysical properties of the nanofluid. 

To increase the accuracy of the model the properties of the nanofluid are modeled as temperature dependent [41]:

(44) $$\rho_{nf}=2263.641-0.636T\;(\mathrm{kg}/m^{3})$$

(45) $$c_{p}=1396.044+0.172T\;(J/\mathrm{kgK})$$

(46) $$\mu_{nf}=0.07543937-2.77\times 10^{-4}T+3.49\times 10^{-7}T^{2}-1.47\times 10^{-10}T^{3}\;(Pa.s)$$

where, T is in Kelvin.

Thermal conductivity of the solar salt is not strongly dependent on the operating temperature as varies between 0.42 and 0.58 W/mK for the temperature range of 600 to 730 K. Therefore, a constant thermal conductivity of 0.45 W/mK is found to be a good choice, showing a 10.12% deviation from the average [41]:

(47) $$k_{nf}=0.45\;\left(\frac{W}{mK}\right)$$

The operational temperature range of the solar salt lies between its melting point of 495 K and boiling point of 873 K [41]. Since, the volume concentration of the nanoparticles was very low in this study, it should have negligible effect on the thermophysical properties of the nanofluid. Nonetheless, a standard Krieger and Dougherty type mixture viscosity model was adopted for the current study:

(48) $$\mu =\mu_{nf}{\left(1-\frac{f_{v}}{\phi_{\max}}\right)}^{-2.5\phi_{\max}}$$

#### 2.4.5. Model Validation

In order to validate this model, it is used to re-create the results of a similar validated model from literature. The model that was re-created was that of Veeraragavan et al. [29], who presented an analytical model for the design of volumetric solar flow receivers using Therminol as the base fluid and graphite as the nanoparticle additive. This particular study was chosen as its model is set up similarly to this study’s model in the respect that it is a 2D parallel plate configuration, the bottom plate is adiabatic, the top plate has thermal losses and the absorbed radiation is modelled as a volumetric heat release. To enable the re-created results to be as similar as possible the same input parameters, boundary conditions and governing equations are used where possible. 

To determine the effect that the receiver length has on the overall efficiency, a parametric sweep of the length was conducted. The sweep ranged from 0.01 m to 0.32 m with a step of 0.01 m was considered. This range equates to a dimensionless length range of 0.123 to 3.94 which is the same as that from the literature. This is determined from equation which defines the dimensionless receiver length:

(49) $$\bar{L}=\frac{L_{rec}}{Pe\cdot y_{rec}}$$

where, *Pe* = Peclet number.

**Figure 4:**
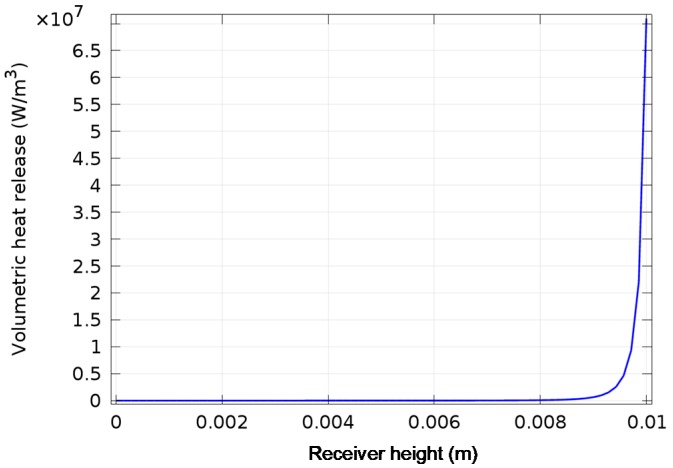
Volumetric heat release profile of receiver.

**Figure 5:**
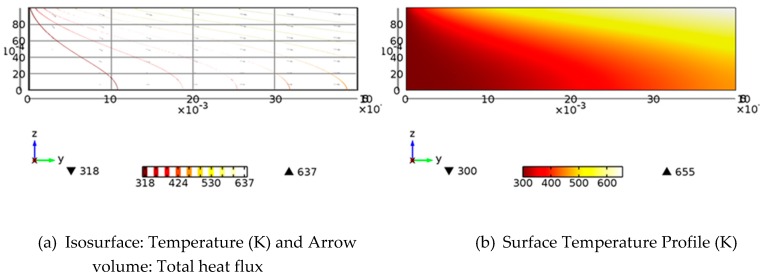
Isothermal contours and temperature profile of the receiver at length 0.4 m.

Figure 4 shows the volumetric heat release profile for the receiver. It illustrates the exponential nature of the attenuation of the solar radiation through the receiver depth. Figure 5 shows the isothermal contours and temperature profile.

**Figure 6:**
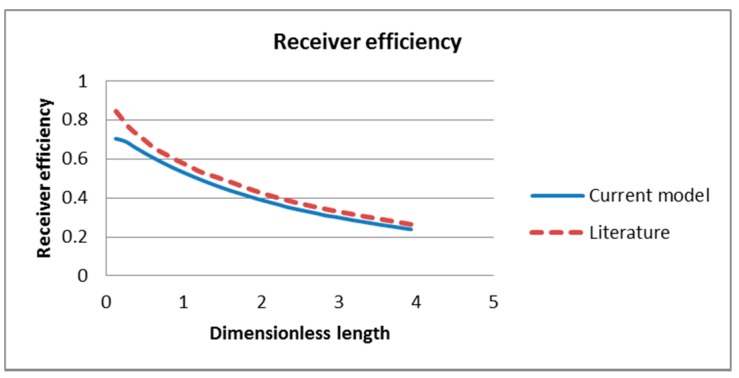
Comparison of receiver efficiency of current model and A. Veeraragavan’s model [29].

**Figure 7:**
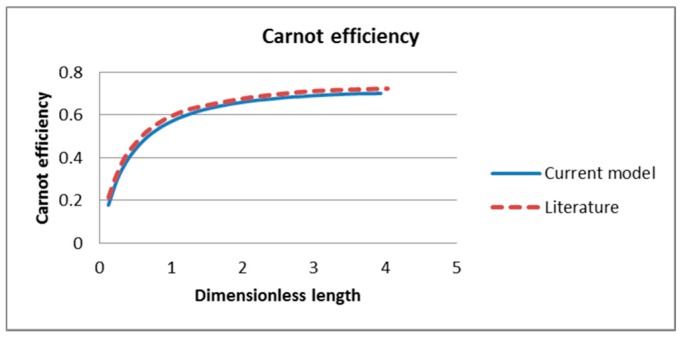
Comparison of Carnot efficiency of current model and literature model [29].

**Figure 8:**
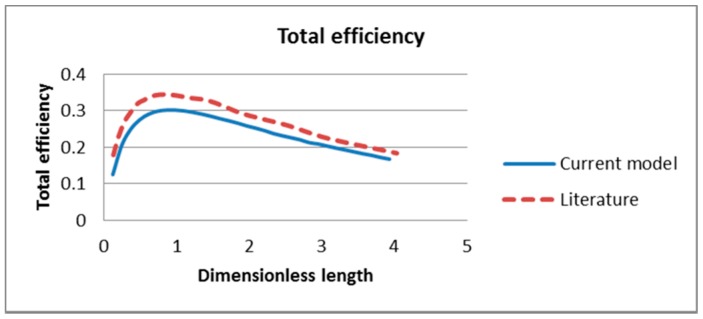
Comparison of total efficiency of current model and literature model [29].

The performance of the receiver is evaluated by three factors: the receiver efficiency, the Carnot efficiency and the total efficiency and compared with those in the literature as shown in Figure 6, Figure 7 and Figure 8 respectively. From the plots it can be seen that there is good agreement between the results as those obtained in this study closely reflect those obtained in the literature. However, this study under-predicts the results slightly for the receiver and Carnot efficiencies. This may be a result of differences in the governing equations used in this study in contrast to an analytical model using simple governing equations and solving combining homogenous and particular integral solutions in the literature. Another factor that may cause the differences in results is the discrepancies in the thermophysical and rheological properties between the two models. The values used for the specific heat, density and viscosity of Therminol in the literature were not stated and therefore this study assumed these values based on other reference [42]:

(50) $$\eta_{tot}=\eta_{rec}\eta_{carnot}=\eta_{rec}\left(1-\frac{T_{amb}}{T_{out}}\right)$$

## 3. Results and Discussion

Due to the acceptable level of agreement between results of current study and Veeraragavan’s model [29], this model can be considered validated. After validation, the model was further improved and several more factors were added. In order to increase the accuracy of the absorptive coefficient of the nanofluid, the model considered the refractive and absorptive indexes of both the base fluid and nanoparticles as wavelength dependent. It also considered initial and inlet temperatures that are significantly higher than that of the ambient temperature. Due to the considerations of these high temperatures, the heat transfer equation is altered to include re-radiation of the nanofluid. The radiative heat loss is defined using Stefan-Boltzmann’s law and the convective heat loss is dependent on the Nusselt number and by default the inlet velocity and base fluid properties. In addition to the convective heat transfer coefficient being dependent on the Nusselt number, this model also considered the thermal resistance of a cover plate; taking into account its thermal conductivity and thickness. Additionally, the nanofluid absorption coefficient is a combination of the base fluid absorption coefficient and the nanoparticle coefficient. 

### 3.1. Effect of Receiver Length and Height on the Efficiencies

The influence of the parameters like receiver length, receiver height, inlet velocity, solar concentration and volume fraction w investigated. The volume fraction was set at 0.00001, height of the receiver was 0.05 m, solar concentration was 10×, ambient temperature was assumed at 297 K and the inlet velocity was set at 0.0001 m/s. To determine the overall power plant efficiency the product of the receiver and power generation system efficiencies are considered.

**Figure 9:**
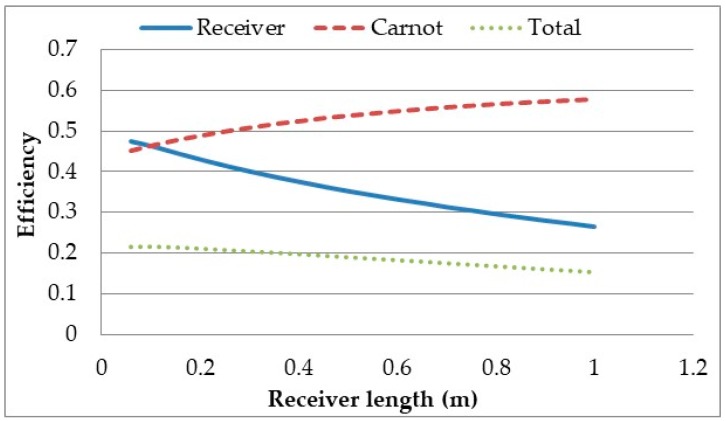
Overall power plant efficiency versus receiver length at high temperature: heat loss calculation was done applying Boltzmann’s law.

This total efficiency represents the maximum achievable efficiency that such a power plant system can theoretically reach. The receiver length was varied between 0.0595 m and 1m for the parameters previously stated and the results are shown in Figure 9. 

**Figure 10:**
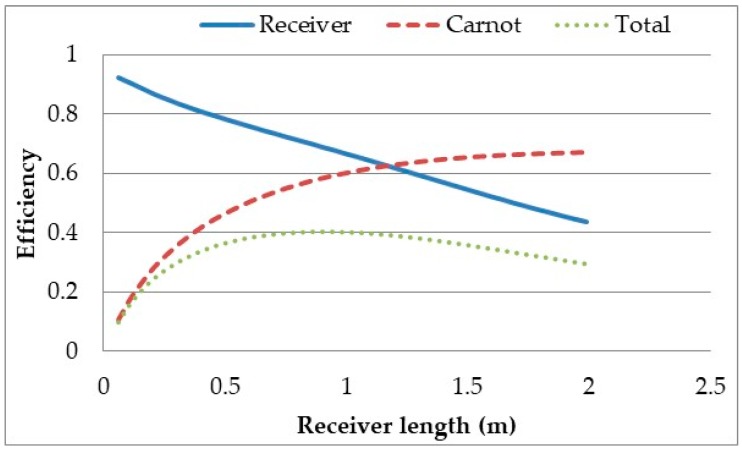
Overall power plant efficiency versus receiver length at high temperature: heat loss calculation applying linear heat loss coefficient.

It can be seen from the results that as the receiver length is increased the receiver efficiency decreases while the Carnot efficiency increases, resulting in a decreasing total efficiency. The Carnot efficiency increases as the longer the receiver is, the longer the heat transfer fluid is exposed to the solar radiation and the greater amount of radiation it is exposed to, resulting in a higher outlet temperature. However, the longer the receiver is the larger the surface area and the hotter the fluid is resulting in greater losses. The plot shows that there does not exist a length where optimal total efficiency is achieved. This result is in contrast with the findings of Veeraragavan et al. [29] who showed that such a length does exist. The discrepancy between the results can be attributed to the higher heat losses in this model due to the use of Boltzmann’s law instead of the linear heat loss coefficient of radiation and high inlet temperature. To check this, the heat loss coefficient for both radiative and convective losses was set to 10W/m^2^K [21] and the inlet temperature set to ambient. It can be seen in Figure 10 that with these altered parameters, there is a length for which the total efficiency is optimised.

**Figure 11:**
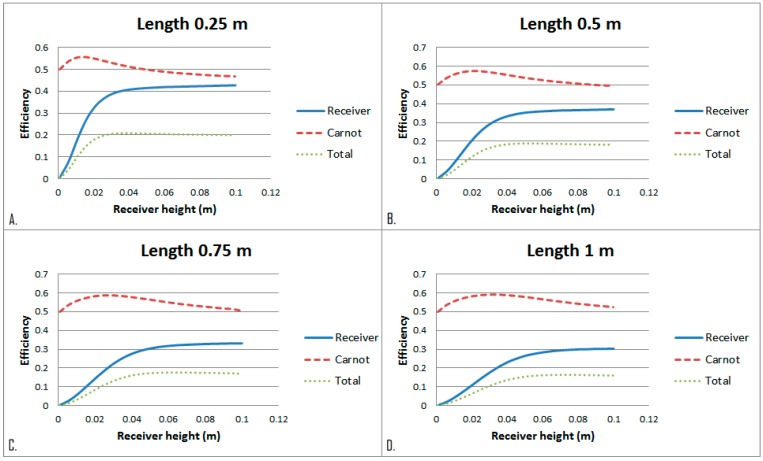
(**A**) Receiver height comparison at length of 0.25 m. (**B**) Receiver height comparison at length 0.5 m. (**C**) Receiver height comparison at length 0.75 m. (**D**) Receiver height comparison at length 1 m.

This implies that for high temperature receivers an optimal length cannot be determined from the overall system efficiency. Instead, other factors need to be considered to determine the optimal length. Effect of receiver height on the efficiencies for a certain length and fixed parameters of the HTF were investigated as shown in Figure 11.

As the depth of the HTF increases with the height of the receiver, larger portion of the incident radiation is absorbed by the bottom surface of the receiver than that of the HTF at shallow depth. This the phenomenon alters gradually with the increase of the receiver height. Therefore, peak Carnot efficiency is achieved at a very low receiver height, while the receiver efficiency is still increasing to a moderate receiver height before levelling off. For total efficiency there is an optimum height for a certain receiver length. 

**Figure 12:**
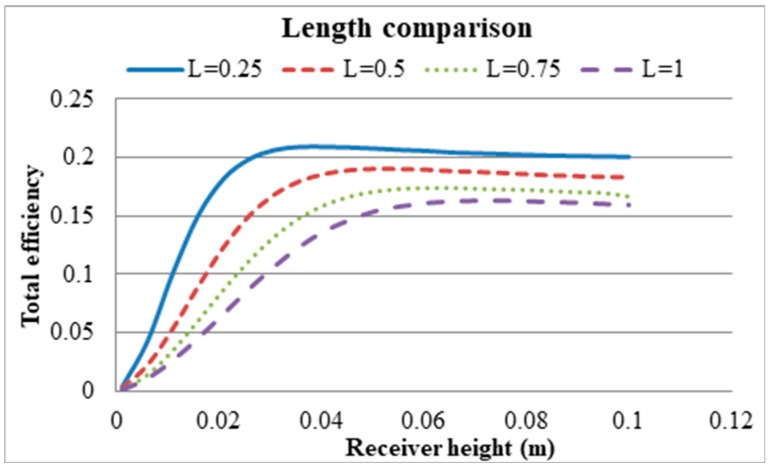
Change of total efficiency (receiver efficiency × Carnot efficiency) with receiver lengths.

**Figure 13:**
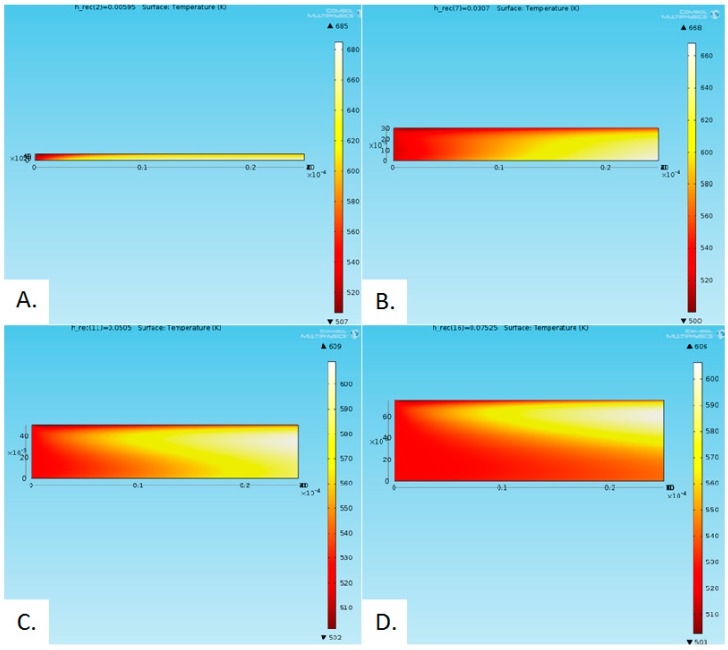
Temperature distribution for different receiver heights: (**A**) 0.00595 m, (**B**) 0.0307 m, (**C**) 0.0505 m and (**D**) 0.07525 m.

From Figure 12 it can also be seen that the peak efficiency decreases with an increase in receiver length, which is in agreement with the results depicted in Figure 11. The peak total efficiencies (receiver efficiency × Carnot efficiency) for different heights and lengths of a receiver are presented in Table 3. As the parametric sweep was conducted with a receiver height step of 0.00495, so, the actual peak may fall within ±0.00495 m of the reported values. Figure 13 shows the temperature distribution at various heights.

### 3.2. Effect of Inlet Velocity on the Efficiencies

**Figure 14:**
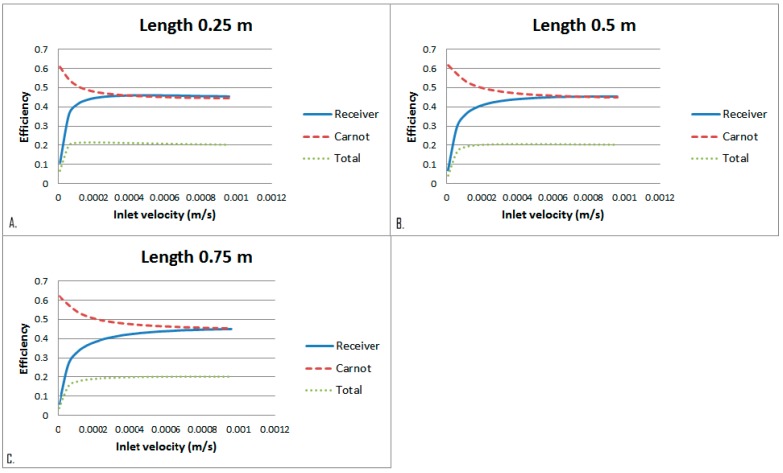
Inlet velocity comparison at different receiver lengths. **A**. 0.25 m, **B**. 0.5 m, **C**. 0.75 m.

Setting the previously defined lengths and their associated optimal heights, the effect of HTF inlet velocity was then investigated. Figure 14 depicts that there is initially a sharp increase in receiver efficiency which then plateaus off quickly. There is then negligible change in total efficiency as the inlet velocity increases. This is a result of the Carnot efficiency decreasing and the receiver efficiency increasing with the inlet velocity. These two balance out and result in an almost constant total efficiency. The Carnot efficiency decreases because the average outlet temperature drops due to the HTF being exposed to the radiation for a less amount of time. The increase in receiver efficiency with inlet velocity is due to the total heat loss being dominated by surface to ambient radiation losses. Even though the convective heat transfer coefficient is increased as the fluid is exposed to the radiation for shorter period of time, causing the temperature to be lower which results in less radiative losses, eventuating in higher receiver efficiency.

**Figure 15:**
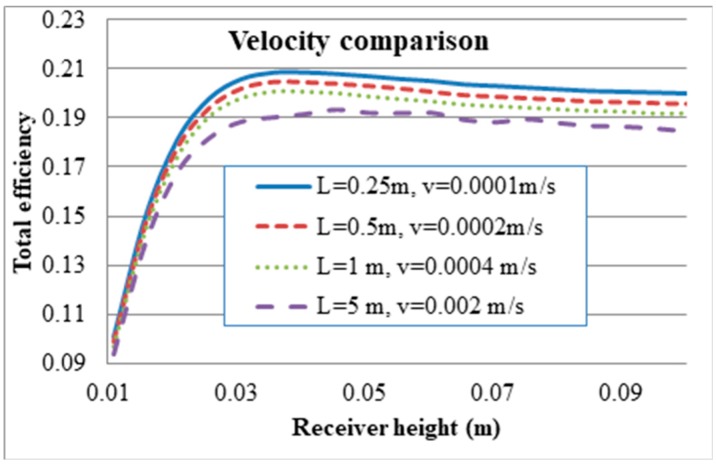
Inlet velocity comparison at different receiver lengths and heights.

To further analyse the effect that the fluid velocity has on the receiver, the dominating effects of surface to ambient radiation need to be considered. To do this the exposure time of the heat transfer fluid is to be kept constant and the inlet velocity varied. This is done by increasing the receiver length so that in all cases the fluid is exposed to the radiation for the same amount of time, arbitrarily chosen as 2500 s. Four cases were considered, a length of 0.25 m at inlet velocity of 0.0001 m/s, length of 0.5 m at inlet velocity of 0.0002 m/s, length of 1m at inlet velocity of 0.0004 m/s and a length of 5 m at inlet velocity of 0.002 m/s. Figure 15 summarises the results and shows that even when the exposure time is constant an increase in velocity results in a decrease in the overall efficiency. This shows that an increase in velocity and by default an increase in Nusselt number results in a higher heat loss coefficient and therefore a decrease in system efficiency.

### 3.3. Effect of Solar Concentrations on the Efficiencies

**Figure 16:**
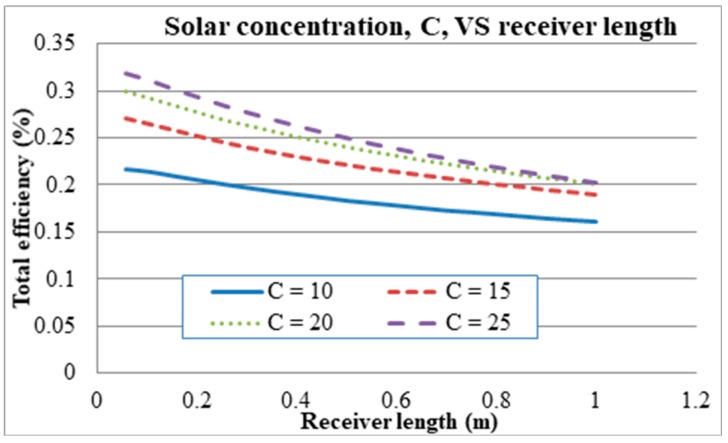
Solar concentration at different receiver lengths.

Setting the volume fraction to 0.00001, the inlet velocity to 0.0001 and the receiver height to 0.0908 m, the solar concentration was then varied for different receiver lengths. The solar concentration was varied from 10×–25× for different receiver lengths as illustrated in Figure 16. The efficiencies are seen increasing with solar concentration but decreasing indefinitely with receiver length.

**Figure 17:**
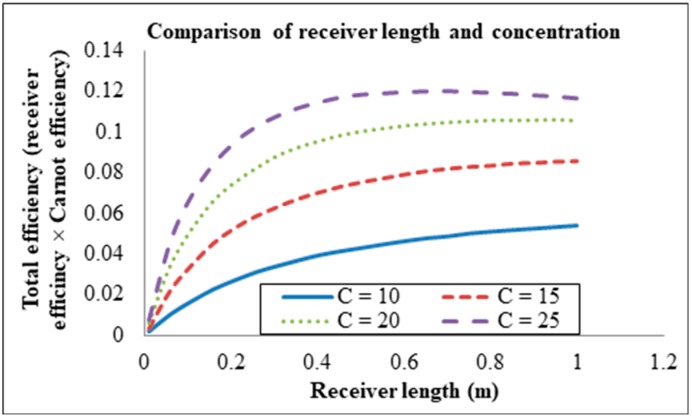
Comparison of receiver length and solar concentration with adjusted Carnot efficiency.

Given that the adjusted Carnot efficiency gives rise to an optimal efficiency for the volume fraction, the receiver length was re-investigated with the adjusted Carnot efficiency as shown in Figure 17.

It can be seen from the plot that a peak in the adjusted efficiency occurs for the receiver length, implying that there exists a length that provides the best trade-off of receiver efficiency for average temperature rise. It can also be seen that the trade-off becomes more pronounced and the optimal receiver length decreases with an increase in solar concentration. Therefore, to achieve the best balance between the receiver efficiency and the temperature rise, short receivers with high solar concentrations are desirable. From this model the suggested optimal design for a solar salt and graphite nanofluid receiver is that consisting of a solar concentration of 25×, volume fraction of 0.00001, inlet velocity 0.0001 m/s, receiver length of 0.406 m and a receiver height of 0.0908 m. These parameters result in an average outlet temperature of 835 K, peak temperature of 870 K, receiver efficiency of 43.9% and a total system efficiency of 28.3%

It should be noted that the model begins to break down at higher concentrations and longer receiver lengths because the viscosity equation results in a negative value due to the high temperatures in those situations. As solar salt begins to break down at 873 K, the maximum temperature should also be considered in the model. To investigate even higher solar concentrations without the viscosity equation breaking down, the inlet velocity is increased while keeping the receiver length constant, allowing for suitable temperatures to be achieved for high concentrations. Table 4 shows the solar concentrations investigated and their respective inlet velocities. 

**Figure 18:**
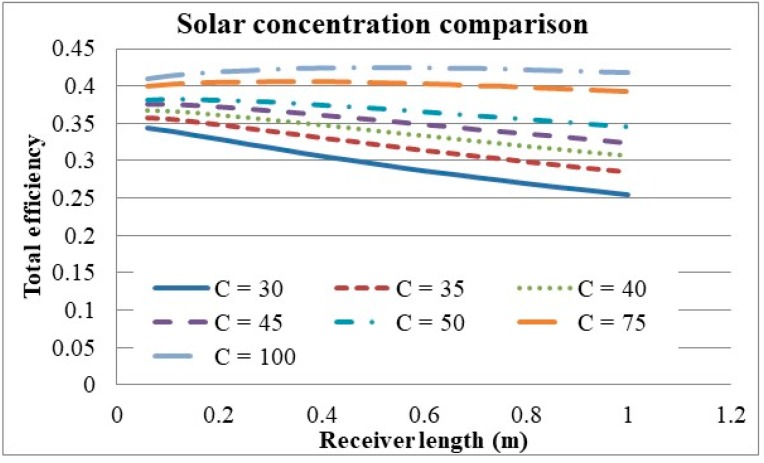
Solar concentration comparison at high concentrations.

The effect that solar concentration has on the total efficiency of the system is illustrated in Figure 18. It can be seen that the total efficiency continually increases with solar concentration and even exceeds efficiencies of 40%. It can also be seen that for higher concentrations, the relationship between the receiver length and total efficiency changes from that of a linear to more of a quadratic relationship, resulting in an optimal length. The reason behind this change can be attributed to the fact that the receiver efficiency becomes less of a dominating factor and the Carnot efficiency has a larger impact on the total efficiency.

**Figure 19:**
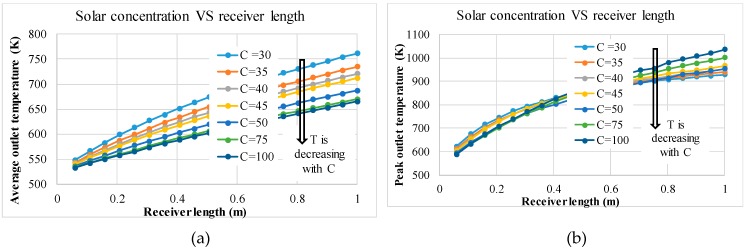
(**a**) Average and (**b**) peak temperatures of the receiver at different receiver lengths and solar concentrations. (In the Figure, T and C are the Kelvin temperature and solar concentration respectively).

However, this plot does not show the peak temperature of the nanofluid. This is shown in Table 5 and it can be seen that after a length of approximately 0.604–0.6535 m, the peak temperature exceeds the upper limit for solar salt. This critical length can be altered to suit operational conditions by varying the inlet velocity; increasing the velocity allows for a longer length and vice versa. From Figure 18, it appears that the more efficient receivers are those with the shortest length. However, in reality, efficiency is not the only factor to be considered. Referring to Table 5 and Figure 19, the shortest receiver lengths result in the lowest outlet temperature, where in the worst case is only 10 K higher than the inlet temperature. This implies that not enough emphasis is paid to the rise in temperature, as it seems that the most efficient receivers are those with the lowest temperature rise.

**Figure 20:**
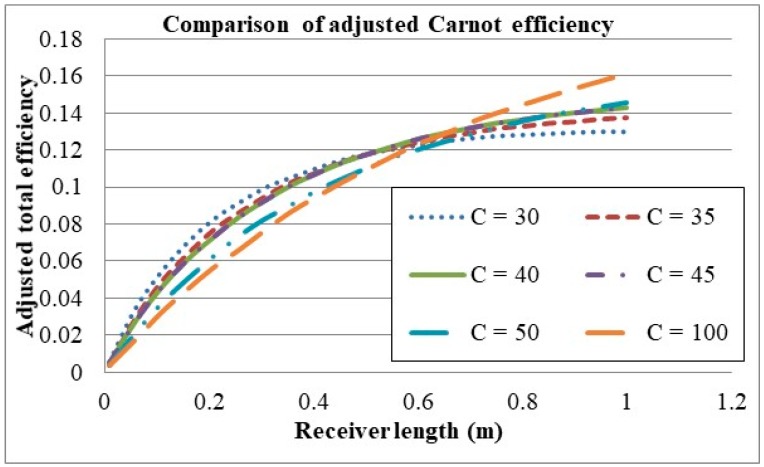
Comparison of solar concentration using adjusted Carnot efficiency.

Considering the adjusted Carnot efficiency, Figure 20 shows which lengths provide the best balance between the receiver efficiency and temperature rise for different concentrations. As no optimal point exists for the receiver length range in question, from the trends presented, it is deduced that the optimal lengths lie beyond those considered range, and the efficiencies increase with increasing of solar concentration. This trend is also represented in Table 5 and Figure 19, which show that the average outlet temperature is lower for high concentrations, with all having similar peak temperatures; meaning that the temperature difference between the average outlet temperature and the peak temperature increases with increasing solar concentration. This is due to the solar radiation being exponentially attenuated by the nanofluid, resulting in the majority of the radiation being absorbed in the upper thin layer of the nanofluid. So an increase in the intensity of the radiation results in an increase in the temperature difference between the peak temperature and average outlet temperature. This is made more evident when comparing solar concentrations of 30× and 100× at conditions where the highest peak temperature is reached without exceeding the upper limit. For a concentration of 30×, a receiver length of 0.5545 m is needed and results in an average outlet temperature of 684 K and a peak temperature of 867 K, resulting in a Carnot efficiency of 57.2%, a receiver efficiency of 49.9% and a total efficiency of 28.5%. In comparison a concentration of 100× results in a receiver length of 0.505 m, an average outlet temperature of 602 K and a peak temperature of 867K, resulting in a Carnot efficiency of 51.2%, a receiver efficiency of 82.8% and a total efficiency of 42.4%.

**Figure 21:**
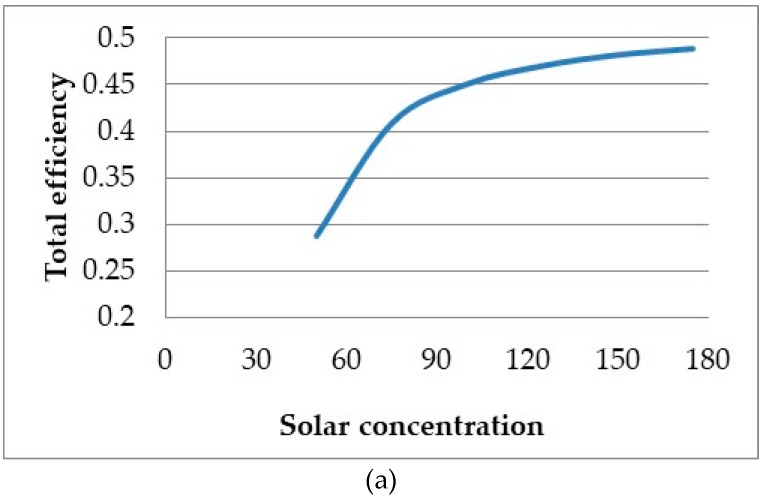

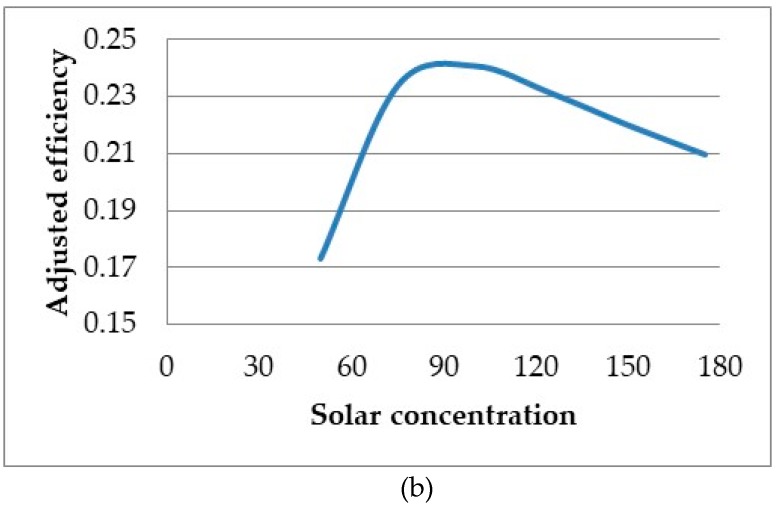
Solar concentration comparison for volume fraction of 0.0005 at a peak temperature of 873 K: (**a**) the total efficiency and (**b**) the adjusted efficiency.

To further investigate the effects of solar concentration, it was varied for each volume fraction and the inlet velocities adjusted such that the peak temperature of the receiver equaled that of solar salt’s upper temperature limit. For a volume fraction of 0.0005 the total efficiency and the adjusted efficiency with respect to solar concentration are depicted in Figure 21a,b respectively.

Figure 21 shows that while the system efficiency increases indefinitely with solar concentration, the adjusted efficiency is peaked approximately at 100× concentration. This better represents the increasing temperature difference between peak and outlet with solar concentration, implying that the best trade-off between receiver efficiency and average temperature rise occurs at a solar concentration of 100×. At higher volume fraction (e.g. 0.0001), most of the energy was absorbed close to the surface so there was a lot of convection and radiation losses due to the high temperature. At lower volume fraction (e.g. 0.00001), the energy was absorbed throughout the receiver with less losses but also resulted in a lower temperature rise in the fluid. However, further increase in volume fraction (e.g. 0.0005) resulted in higher total efficiency. Higher absorption of heat due to higher volume fraction was much higher than the loss to the environment. 

**Figure 22:**
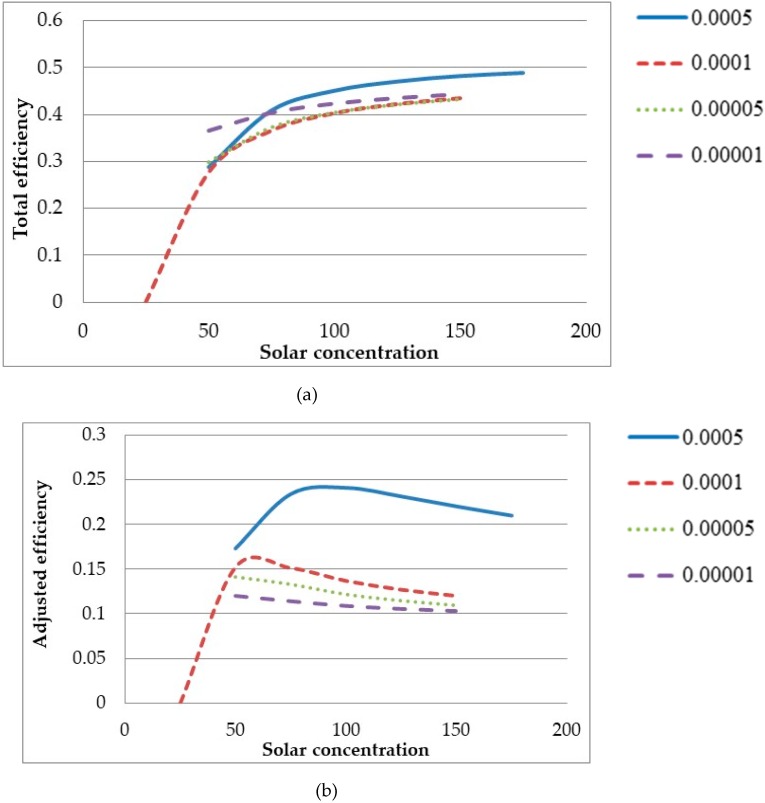
Solar concentration comparison at different volume fractions: (**a**) the total efficiency and (**b**) the adjusted efficiency (The legend at the right side of the figure shows the volume fractions).

**Figure 23:**
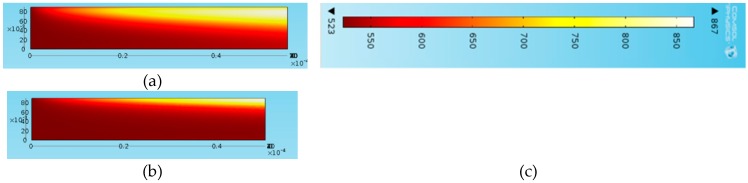
Temperature distribution at different solar concentrations: (**a**) solar concentration, C = 30×, flow velocity, v = 0.0002 m/s, (**b**) C = 100×, v = 0.002 m/s and (**c**) the colour scheme shows the temperature scale.

The same simulations were conducted for different volume fractions and the results are illustrated in Figure 22. With increasing solar concentration, each volume fraction shows a similar trend in Figure 22a in a sense that the total efficiency increases indefinitely, and the adjusted efficiency is peaked between 50× and 100× concentration at first then starts to decrease. In addition, it can be seen from Figure 22b that the peak solar concentration decreases with decreasing volume fraction such that it is about 100× for a volume fraction of 0.0005 and then approximately 50× for the remaining volume fractions. This can be attributed to the fact that, similar to solar concentration, a decrease in volume fraction results in an increase in the temperature difference between the peak and average outlet temperatures. So with the decreasing volume fraction and increasing solar concentration both increasing the temperature difference. This results in a lower adjusted efficiency and by extension reducing the peak solar concentration. Figure 23 illustrates temperature profile at two different solar concentrations. It can be seen that the lower concentration has a more even temperature distribution compared to the higher concentration.

## 4. Conclusions

Nanofluid volumetric flow receivers have been studied for high-temperature concentrating solar thermal applications using sodium nitrate + potassium nitrate salts, NaNO_3_-KNO_3_, also commonly known as solar salt base fluids doped with graphite. A 2D CFD model is developed to investigate the effects of design and operating parameters on the receiver efficiency, temperature rise and system efficiency. Parameters taken into consideration included receiver length, inlet velocity and solar concentration. The model has been evaluated using three types of heat losses from the nanofluid: surface to ambient radiation loss, convective loss and re-emission loss. 

The model shows that the receiver efficiency increases with increasing solar concentration, decreasing receiver length and decreasing nanoparticle volume fraction and, by extension, increasing receiver height. It also showed that the temperature rise across the receiver increases with an increase in receiver length, decrease in inlet velocity and an increase in solar concentration. When the receiver is connected to a power generation cycle, the total system efficiency is found to be in excess of 40% when solar concentrations are greater than 100×. 

The reason behind the high concentration receivers resulting in a higher efficiency is that the increase in receiver efficiency outweighs the decrease in Carnot efficiency, as the receiver efficiency is found to exceed 90% in certain cases. It was also revealed that an optimal receiver length exists which increases with increasing solar concentration, and is also dependent on the inlet velocity. In addition, adjusted total efficiency resulted in a peak for solar concentration, which decreased with decreasing volume fraction, implying that each receiver configuration has an optimal solar concentration. 

This study provides a comprehensive model of a direct absorption high temperature nanofluid solar receiver which can be used to determine an optimal receiver design given the operating conditions of the system.

## Figures and Tables

**Table 1:** Parameters used in the computational model.

Name	Expression	Description
$y_{rec}$	0.01 (m)	Height of receiver
$w_{rec}$	0.001 (m)	Width of receiver
$l_{rec}$	0.04 (m)	Length of receiver
$k_{bf}$	0.135 (W/mK)	Thermal conductivity of base fluid
$C_{p,bf}$	1575 (J/kgK)	Specific heat of base fluid
$\rho_{bf}$	1056 (kg/m^3^)	Density of base fluid
$\mu_{bf}$	0.00328 (Pa.s)	Dynamic viscosity of base fluid
$\rho_{P}$	2100 (kg/m^3^)	Density of nanoparticles
$T_{in}$	300 (K)	Inlet temperature
$\nu_{in}$	0.000066 (m/s)	Inlet velocity
D	50 × 10^−9^ (m)	Diameter of nanoparticles
$V_{f,P}$	0.000634	Volume fraction of nanoparticles
$S_{att}$	0.73	Attenuation constant
Conc	10×	Solar concentration (times)
$\Omega_{S}$	6.8 × 10^−5^ (steradians)	Solid angle of the sun from Earth
h	6.62606957 × 10^−34^ (J.s)	Planck’s constant
$K_{b}$	1.3806488 × 10^−23^ (J/K)	Boltzmann constant
$T_{sun}$	5800 (K)	Temperature of the sun
C	299792458 (m/s)	Speed of light
$T_{amb}$	$T_{in}$	Ambient temperature
$n_{P}$	2.72	Refractive index of nanoparticle
$k_{abs,P}$	1.31	Absorptive index of nanoparticle
$n_{bf}$	1.63	Refractive index of base fluid
$k_{abs,bf}$	3.86 × 10^−8^	Absorptive index of base fluid
$\lambda_{min}$	100 × 10^−9^ (m)	Lower limit of wavelength range
$\lambda_{max}$	1000 × 10^−6^ (m)	Upper limit of wavelength range
$h_{total}$	13.5 (W/m^2^K)	Combined radiative and convective heat loss coefficient

**Table 2:** Variables used in the computational model.

Name	Expression	Description
λ	$range\left(\lambda_{\min},\lambda_{\max}\right)$	Wavelength
$I_{0}$	$S_{att}{C\Omega }_{s}\frac{2h_{P}C^{2}}{\lambda^{5}}\frac{1}{e^{\frac{hC}{\lambda \kappa_{B}T_{sun}}}-1}$	Concentrated normally incident solar radiation distribution
$P_{y,0}$	∫λminλmaxI0dλ	Concentrated normally incident solar radiation
$y_{1}$	$y_{rec}-Z$	Distance from surface of receiver
$m_{nf}$	$\frac{n_{P}+ik_{abs,P}}{n_{bf}+ik_{abs,bf}}$	Relative complex refraction index of nanofluid
*M*	$\frac{m_{nf}^{2}-1}{m_{nf}^{2}+2}$	Total mass of nanofluid
$M_{2}$	$\frac{m_{nf}^{4}+27m_{nf}^{2}+38}{2m_{nf}^{2}+3}$	
$Q_{abs}$	$\frac{4\pi D}{\lambda }Im\left[M\left\{1+{\left(\frac{\pi D}{\lambda }\right)}^{2}\frac{1}{15}MM_{2}\right\}\right]$	Absorption efficiency
$Q_{scat}$	$\frac{8}{3}{\left(\frac{\pi D}{\lambda }\right)}^{4}{\left|M\right|}^{2}$	Scattering efficiency
$\kappa_{abs}$	$\frac{3V_{f,P}\left(Q_{abs}+Q_{scat}\right)}{2D}$	Absorption coefficient of nanoparticles
$\kappa_{abs,bf}$	$\frac{4\pi \kappa_{abs}}{\lambda }$	Absorption coefficient of base fluid
$I_{y}$	$I_{0}e^{-\kappa_{abs,total}y_{1}}$	Spectral flux
$P_{y}$	∫λminλmaxIydλ	Divergence of the spectral flux
$Q_{source}$	$-\frac{dP_{y}}{dy_{1}}$	Volumetric heat release
$T_{ave,out}$	ave,out(T)	Average outlet temperature
$\eta_{rec}$	$\frac{\rho_{bf}\nu_{in}y_{rec}w_{rec}C_{P,bf}(T_{out}-T_{in})}{P_{y,0}l_{rec}w_{rec}}$	Efficiency of receiver

**Table 3:** Receiver height comparison at different lengths.

Receiver Length (m)	Peak Height (m)	Total Efficiency
0.25	0.0406	20.9%
0.5	0.0505	19%
0.75	0.06535	17.5%
1	0.07525	16.3%

**Table 4:** Inlet velocities for solar concentrations.

Solar Concentration (×)	Inlet Velocity (m/s)
30	0.00020
35	0.00030
40	0.00040
45	0.00050
50	0.00070
75	0.00135
100	0.00200

**Table 5:** Average and peak temperatures of the receiver at different receiver lengths and solar concentrations. Temperatures are in Kelvin and values exceeding the upper limit of the nanofluid are in red.

Conc.	30		35		40		45		50		75		100	
Temp.	Ave.	Peak	Ave.	Peak	Ave.	Peak	Ave.	Peak	Ave.	Peak	Ave.	Peak	Ave.	Peak
L. (m)														
0.0595	548	624	543	613	541	608	540	605	536	594	534	589	533	588
0.109	566	676	559	663	555	656	553	654	547	639	543	633	542	633
0.1585	583	716	573	702	568	698	565	696	557	676	552	669	550	673
0.208	599	745	587	735	580	731	576	730	567	709	560	702	558	709
0.2575	613	773	599	760	592	759	588	759	577	737	568	735	565	739
0.307	627	794	611	783	603	783	598	784	586	763	576	763	573	770
0.3565	639	812	623	804	614	805	608	807	594	786	584	788	581	797
0.406	651	830	633	825	624	824	618	823	603	802	592	814	588	825
0.4555	663	843	644	840	634	840	627	841	611	820	599	836	595	848
0.505	674	855	654	851	643	853	636	861	619	841	606	855	602	867
0.5545	684	867	663	865	652	868	645	871	627	854	613	876	609	892
0.604	694	876	672	879	661	882	653	887	634	867	620	893	616	914
0.6535	704	886	681	888	669	893	662	900	641	881	627	909	622	932
0.703	713	893	689	898	677	905	669	913	648	893	633	925	629	948
0.7525	722	901	698	906	685	913	677	920	655	905	640	938	635	956
0.802	730	908	705	913	692	923	684	933	662	915	646	954	641	981
0.8515	738	912	713	920	700	931	692	942	669	930	652	966	647	995
0.901	746	919	720	927	707	938	699	948	675	935	658	977	653	1008
0.9505	754	924	728	935	714	945	705	956	681	944	664	988	659	1021
1	761	929	735	939	721	953	712	966	687	954	670	1001	665	1036

## Nomenclature

$A_{r}$	Collector area (m^2^).
*a_o_* and *a*_1_	Constant parameters.
$a_{nP}$	Absorptive index.
C	Concentration factor (×).
c	Speed of light (m/s).
$C_{P}$	Specific heat (W/mK).
D	Particle diameter (m).
$f_{v}$	Volume fraction of nanoparticles.
h	Planck’s constant.
$h_{total}$	Overall heat transfer coefficient (W/mK).
$h_{nf}$	Nanofluid heat transfer coefficient (W/mK).
$I_{0}$	Incident radiation (W/m^2^).
$I_{s}$	Scattered irradiation (W/m^2^).
k	Thermal conductivity (W/m^2^K).
$k_{b}$	Boltzmann constant.
$k_{nf}$	Thermal conductivity of nanofluid (W/m^2^K).
$k_{tp}$	Thermal conductivity of top plate (W/m^2^K).
L	Characteristic length taken as receiver height (m).
$y_{rec}$	Length of receiver (m).
m	Relative complex refractive index of the nanofluid= *S_np_* / *S_bf_*.
$\dot{m}$	Mass flow rate (kg/s).
N	Number of scattering particles in the beam path.
$N_{u}$	Nusselt number.
$n_{np}$	Refractive index.
$P_{0}$	Solar irradiance integrated over solar wavelength range (W/m^2^).
Pr	Prandtl number.
Q	Heat source (W).
Re	Reynolds number.
$S_{att}$	Attenuation constant: which accounts for the average attenuation through Earth’s atmosphere.
$S_{bf}$	Complex refractive index of the base fluid.
$S_{j}$	Strength of the jth resonant vibration mode.
$S_{np}$	Complex refractive index of the nanoparticles.
T	Temperature (K).
$T_{amb}$	Ambient temperature (K).
$T_{in}$	Inlet temperature (K).
$T_{out}$	Average outlet temperature (K).
$T_{sun}$	Temperature of the sun (K).
t	Thickness of top plate (m).
u	Fully developed velocity (m/s).
$\nu_{in}$	Inlet nanofluid velocity (m/s).
**Greek symbols**	
ϑ	Scattering angle.
$\Omega_{s}$	Solid angle of the sun as seen from Earth (steradian).
$\delta_{j}$	Damping parameter of the jth resonant vibration mode.
$\phi_{\max}$	Maximum packing concentration.
$\eta_{rec}$	Receiver efficiency (%).
$\eta_{Carnot}$	Carnot efficiency (%).
$\eta_{tot}$	Total efficiency (%).
λ	Radiation wavelength (m).
$\lambda_{j}$	Characteristic wavelength of the jth resonant vibration mode (m).
θ	Incident angle (rad).
ρ	Density (kg/m^3^).
